# BRCA1 and BRCA2 mutations and their clinical relevance in selected women diagnosed with triple-negative breast cancer in Kenya: a descriptive cross-sectional study

**DOI:** 10.11604/pamj.2023.45.102.36431

**Published:** 2023-06-23

**Authors:** Josephine Nyabeta Rioki, Lucy Muchiri, Marshal Mweu, Joshua Nyagol, Elijah Songok, Joseph Mwangi, Micah Oyaro, Laban Bwire Ong'ang'o, Emily Rogena

**Affiliations:** 1 Department of Human Pathology, Faculty of Health Sciences, University of Nairobi, Nairobi, Kenya,; 2 Department of Public and Global Health, Faculty of Health Sciences, University of Nairobi, Nairobi, Kenya,; 3 Kenya Medical Research Institute (KEMRI), Nairobi, Kenya,; 4 Massive Genomics solutions, Nairobi, Kenya,; 5 Department of Pathology, Jomo Kenyatta University of Agriculture and Technology, Nairobi, Kenya

**Keywords:** Genes, BRCA1, BRCA2, triple-negative breast neoplasms, pathogenic mutation

## Abstract

**Introduction:**

triple-negative breast cancer (TNBC) is a heterogeneous breast cancer type with a poor prognosis. About 25% of TNBC patients carry breast cancer susceptibility genes 1 and 2 (BRCA1 and BRCA2) mutations. Screening for BRCA mutations would facilitate early detection and initiation of personalized therapy, thus improving prognosis. However, this has not been explored in our population. We aimed at identifying BRCA1 and BRCA2 gene mutations and their clinical relevance among selected women with TNBC in Kenya.

**Methods:**

six participants enrolled in a larger descriptive cross-sectional study who met the inclusion criteria were selected. Structured questionnaires were used to obtain qualitative data. Deoxyribonucleic acid (DNA) was extracted from saliva. Whole exome sequencing of BRCA1 and BRCA2 genes using a next-generation sequencer was done.

**Results:**

overall, 83.3% of BRCA1 and BRCA2 gene mutations with clinical relevance were detected. Most of the variants (63%) were found in BRCA1 whereas 37% were found in BRCA2. Pathogenic mutations in BRCA1 gene included c.5513T>A, c.5291T>C, c.5297T>G, c.110C>A, c.5212G>C, c.122A>C, c.5117G>A, c.5095C>T, c.5054C>T, c.5053A>G, c.115T>A, c.5143A>G, and c.130T>G. Those in BRCA2 gene were c.7878G>A, c.9154C>T, c.8243G>A, c.7976G>A, c.8165C>G, c.8167G>C, and c.8168A>T. One variant (c.5352delG: p. Leu1785Terfs) not matching any in the BRCA Exchange and ClinVar databases was detected.

**Conclusion:**

our study revealed BRCA mutations that could be common among our population. Further, it has shown that BRCA1 and BRCA2 genetic mutations identified are of clinical relevance and there is a need to screen for these mutations in breast cancer patients to understand their implication in patient management outcomes.

## Introduction

Breast cancer (BC) is the commonest form of malignant tumor among women worldwide. It is a major public health concern in both low and middle-income countries [1]. Patients usually present late and consequently, most of them are diagnosed with advanced stage of the disease, complicating the management process. In Africa including Kenya, the situation is similar. An estimated six thousand (6,000) new cases and 2,500 cancer-related deaths occur per year in Kenya [2]. Breast cancer is a heterogeneous disease exhibited by different types. Triple-negative breast cancer (TNBC) is one of the most aggressive subtypes of breast cancer. It lacks estrogen (ER) and progesterone (PR) receptors and human epidermal growth factor (HER 2) expression or amplification. It is characterized by early-onset (<50 years), aggressive behavior, a high risk of recurrence, metastasis, and poor survival [3,4]. This cancer represents 14-16% of all breast cancers. About 25% of TNBC patients carry germline breast cancer gene types 1 and 2 (BRCA1 and BRCA2) mutations [5]. Generally, carriers of BRCA1 and BRCA2 mutations have an increased risk (71.4-87% for the BRCA1 mutation and 77-88% for the BRCA2 mutation) of developing breast and ovarian cancers [6]. Although this necessitates genetic testing for cancer patients and the population at risk [7,8], this is not a common practice in many setups in developing countries because of the high cost of testing. Genetic testing would facilitate the identification of those who can benefit from targeted therapies, mainly poly adenosine diphosphate-ribose polymerase (PARP) inhibitors [9]. Triple negative breast cancer patients can also opt for contralateral prophylactic mastectomy and oophorectomy once their risk is determined [10]. For relatives of patients with germline mutations, BRCA testing would enable the identification of those at high risk of early onset of cancer, enhancing cancer prevention strategies such as increased surveillance. A number of studies have documented the spectrum and association between BRCA gene mutations and breast cancer patients including those with TNBC. This has informed genetic screening of women with breast cancer. However, genetic risk assessment of BRCA gene mutations in the Kenyan population remains unexplored, yet, we have an upsurge in the early onset of breast cancer. In addition, there is limited accessibility to genetic testing [11]. In this study, we sought to screen for BRAC1 and 2 genetic mutations among women with TNBC attending selected referral hospitals in Kenya, to inform on their spectrum and potential clinical utility in the management of breast cancer patients.

## Methods

**Study design and recruitment of participants:** in this descriptive cross-sectional study, we enrolled selected women from a breast cancer prospective study cohort in Kenya. Detailed information about the study, risks, and benefits was explained, and consent was sought from them. All the participants gave written informed consent to undergo genetic testing. Genetic counseling was done before specimen collection. This study was carried out in one of the major teaching and referral facilities in Nairobi, Kenya, between 2016 and 2021. The eligibility criteria included positive for triple-negative breast cancer and willingness to give written informed consent. Six (6) participants who met these eligibility criteria from the prospective study were purposively selected to participate in this study. Demographic data which included age and ethnicity, and clinical data such as age at first diagnosis, stage, and grade of cancer, and family history of breast and other cancers were collected using structured questionnaires. Deoxyribonucleic acid (DNA) sequencing was done to determine if the participants had mutations. These were further classified as pathogenic, likely pathogenic, those of conflicting interpretations and variants of undetermined significance.

**Specimen collection and storage:** saliva was collected from six patients with TNBC using non-invasive sample collection devices [ORAgene. DNA (OG-500)-DNA Genotek Inc. Ottawa ON, Canada K2K1L1 kit]. The devices were labeled with study numbers to assure confidentiality. Collected samples were stored at room temperature as per the manufacturer´s instructions till use.

### Laboratory procedures

**Genomic DNA extraction and testing for BRAC1 and BRCA2 mutations using next-generation sequencing:** genomic DNA was extracted using the iPrep™ PureLink® gDNA Blood Kit by Life Technologies. Extracted DNA samples were checked for purity and integrity using a NanoDrop 1000 system (Thermo fisher scientific) and for concentration using a Qubit 3.0 fluorometer (Thermo fisher scientific).

**Library preparation and Ion S5 XL sequencing:** the Oncomine™ panel used for library preparation covered 100% of the coding sequences of BRCA1 and BRCA2, including all splice sites with an average of 64bp extensions from the intron junctions. Library preparation and sequencing were done using a protocol described by Yoo *et al*. 2020 without any modification [12]. Overall, library preparation was done using an ion chef system (Thermo Fisher Scientific) according to the manufacturer´s instructions. Briefly, barcoded libraries were generated from 10ng of DNA per sample using an ion ampliSeq chef solutions DL8 Kit (Thermo fisher scientific) and an Oncomine™ BRCA research assay (Thermo fisher scientific). Two pre-mix pools of 265 primer pairs were used to generate sequencing libraries. Clonal amplification of the libraries was carried out by emulsion polymerase chain reaction (PCR) using an Ion Ampliseq IC 200 Kit (Thermo fisher scientific). The prepared libraries were sequenced on an Ion S5 XL sequencer using an Ion 520 Chip and an Ion 520 kit -chef kit (all Thermo Fisher Scientific) [12].

**Bioinformatic analyses:** raw sequence data was generated in FASTQ format and aligned to hg19 human reference genome using the Torrent Mapping Alignment Program aligner implemented in v.5.2 of the Torrent suite software (Thermo fisher scientific). The Torrent variant caller v.5.2.0.34 (Thermo fisher scientific) was used for single nucleotide variant (SNV) calling to generate a variant call format file. With the Torrent variant caller analysis, the default setting of germline low-stringency parameters (minimal variant frequency of 0.1, minimum variant quality of 10, minimum coverage of 5X, maximum strand bias of 0.98, and a minimum variant score of 10) was used. Candidate variants were obtained only when variant frequency reached a given position of ≥ 20% and variant coverage of ≥ 20X. Variant calls from the Torrent variant caller were visualized by the Oncomine^TM^ reporter software online (Thermo fisher scientific) and the integrative genomics viewer software (Broad Institute, Cambridge, MA, USA) [12].

**Data analysis:** analysis of data was done on R (version 4.0.5). Only variants that passed the laboratory filtering technique (i.e. filter=Pass) were considered viable for analysis. To establish the clinical significance of the variants, a search in the BRCA Exchange and ClinVar databases was done.

**Ethical approval and selection of the study participants:** ethical approval to conduct the study was obtained from Kenyatta National Hospital- UoN Ethics and Research Committee (KNH-UoN ERC), reference number P334/04/2016. Written informed consent to participate in the study was obtained.

## Results

In the main study, 145 study participants had breast cancer. However, six of them confirmed to have triple-negative breast cancer were enrolled in this study. The age range of the study participants was 16-52 years, with a median age of 47 years. Three patients (50%) reported a history of cancer in their families. Most of the patients had grade 3 and stage 3b breast tumors. The age at diagnosis of one participant was < 20 years, three participants were <40 years, while the rest were at >40 years. The tumors were located mostly on the upper inner quadrants of the left breast. All six patients had invasive ductal carcinoma, grade 3 tumors. Full demographic and clinicopathological details are included in Table 1. In all the study participants, missense mutations were identified. Forty-eight (48) pathogenic mutations and five variants of undetermined significance (VUS) were detected both in the coding regions and splice sites of the BRCA gene. Of these mutations, 43 were shared among 5 study participants (Table 2, Table 3). One of the study participants had a variant (c.5352delG: p. Leu1785Terfs) on exon 21 of BRCA1 that was not matching to any variant in the BRCA1 and BRCA2 Exchange, and ClinVar databases. This variant has previously been reported in a conference proceeding (Sri Lankan cohort, 2019). Another study participant (JR02) had a mutation in exon 11 of BRCA1 (c.4547G>T: p. Arg1516Met) which was not shared among the other study participants. Five (5) VUS were detected in our study. In BRCA 2, c.7879A>G and c.7988A>G were found in exons 17 and 18, while in BRCA 1, c.5123C>T, c.5324T>C, and c.4900A>G were found in exons 18, 21 and 16. Whereas most of the mutations were shared among 5 study subjects, there were three mutations (c.5291T>C, c.5297T>G, and c.4900A>G) in BRCA1, and one (c.9154C>T) in BRCA2, that were only shared among two, three and four participants. In BRCA1, c.5291T>C was shared among JR01, JR03, JR 05, and JR06, c.5297T>G was shared among JR01 and JR06, and c.4900A>G was shared among JR05 and JR06. In BRCA2, c.9154C>T was shared among JR01, JR04, and JR06. Pathogenic mutations were the most identified (68.8%; n=33/48), followed by Pathogenic/Likely pathogenic (14.6%; n=7/48), VUS (10.4%; n=5/48), with those with conflicting interpretations of pathogenicity and likely pathogenic being 2.1% (n=1/48) each. Most of the variants (63%) were found in BRCA1 whereas (37%) were found in BRCA2. Total variants identified in the exons of BRCA1 were 21, translating to 70% (n=21/30), while 30% (n=9/30) were those found in exons of BRCA2. The proportion of variants in the splice sites of BRCA1 was 52.6% (n=10/19), while 47.4% (n=9/19) were those found in the splice sites of BRCA2. The majority (22.7%) of the variants were found in exon 18 of BRCA2 followed by 18.2% in exon 14 and 17, 10% in exon 16, and the rest had 4.5% each. In BRCA1, 19.4% of the variants were found in exon 21 followed by 16.1% in 3 and 18, 9.7% in exon 17, and 6.7% in exon 11 while the rest had 3.3% each.

**Table 1:** participants' demographics and clinicopathological characteristics among triple-negative breast cancer patients (n=6)

Participants	JR01	JRO2	JR03	JR04	JR05	JR06
Age range at recruitment (years)	35-44	35-44	15-24	35-44	45-54	45-54
Age range at diagnosis (years)	35-44	35-44	<15	35-44	45-54	45-54
Ethnicity	Bantu	Bantu	Bantu	Kalenjin	Bantu	Bantu
Laterality of breast	Left	Left	Left	Right	Left	Right
Family history of cancer	Liver	Breast	None	None	Liver, pancreas, prostate, skin	None
Tumor stage	Stage IA	Stage IIIB	Stage IIIB	Stage IIIB	Stage IIIB	Stage IIB

**Table 2:** genotypic mutations on exons of BRCA1 and BRCA2 among the participants and their clinical significance (n=5)

Gene	Genotype	Amino acid change	Exon	REF	ALT	dbSNP	Clinical significance
BRCA1	c.5513T>A	p.Val1838Glu	24	A	T	rs80357107	Pathogenic
	c.5291T>C	p.Leu1764Pro	21	A	G	rs80357281	Pathogenic
	c.5297T>G	p.Ile1766Ser	21	A	C	rs80357463	Pathogenic
	c.110C>A	p.Thr37Lys	3	G	T	rs80356880	Pathogenic
	c.5212G>C	p.Gly1738Arg	20	C	T	rs80356937	Pathogenic
	c.122A>C	p.His41Pro	3	T	C	rs80357276	Pathogenic
	c.5117G>A	p.Gly1706Glu	18	C	T	rs80356860	Pathogenic
	c.5095C>T	p.Arg1699Trp	18	G	A	rs55770810	Pathogenic
	c.5054C>T	p.Thr1685Ile	17	G	A	rs80357043	Pathogenic
	c.5053A>G	p.Thr1685Ala	17	T	C	rs80356890	Pathogenic
	c.181T>C	p.Cys61Arg	4	A	C	rs28897672	Pathogenic/ likely pathogenic
	c.131G>T	p.Cys44Phe	3	C	T	rs80357446	Pathogenic
	c.115T>A	p.Cys39Ser	3	A	G	rs80357164	Pathogenic/ likely pathogenic
	c.5143A>G	p.Se1715Arg	18	T	G	rs80357222	Pathogenic
	c.130T>G	p.Cys44Gly	3	A	T	rs80357327	Pathogenic
	c.5359T>A	p.Cys1787Ser	22	A	T	rs80357065	Conflicting interpretations of pathogenicity
	c.5324T>C	p.Met1775Thr	21	A	C	rs41293463	Uncertain significance
	c.5123C>T	p.Ala1708Val	18	G	T	rs28897696	Uncertain significance
	c.4900A>G	p.Arg1634Gly	16	T	C	rs1597830733	Uncertain significance
BRCA2	c.7878G>A	p.Trp2626Ter	17	G	C	rs80359013	Pathogenic
	c.9154C>T	p.Arg3052Trp	24	C	T	rs45580035	Pathogenic
	c.8243G>A	p.Gly2748Asp	18	G	A	rs80359071	Pathogenic
	c.7976G>A	p.Arg2659Lys	17	G	C	rs80359027	Pathogenic
	c.8165C>G	p.Thr2722Arg	18	C	G	rs80359062	Pathogenic
	c.8167G>C	p.Asp2723His	18	G	C	rs41293511	Pathogenic
	c.8168A>T	p.Asp2723Val	18	A	G	rs41293513	Pathogenic/likely pathogenic
	c.7988A>G	p.Glu2663Gly	18	A	T	rs80359031	Uncertain significance
	c.7879A>G	p.Ile2627Val	17	A	T	rs80359014	Uncertain significance

**Table 3:** genotypic mutations on splice-sites of BRCA1 and BRCA2 among the participants and their clinical significance (n=5)

Gene	Genotype	Exon	REF	ALT	dbSNP	Clinical significance
BRCA1	c.5332+1G>C	21	C	T	rs80358041	Pathogenic/likely pathogenic
	c.5278-2del	21	CT	C	rs878853285	Pathogenic
	c.5153-1G>T	19	C	G	rs80358137	Pathogenic
	c.5152+1G>A	18	C	A	rs80358094	Pathogenic
	c.5074+1G>T	17	C	T	rs80358053	Pathogenic
	c.4675+1G>C	15	C	T	rs80358044	Pathogenic
	c.4357+1G>C	12	C	T	rs80358027	Likely pathogenic
	c.4097-1G>A	11	C	T	rs80358070	Pathogenic
	c.547+2T>A	7	A	T	rs80358047	Pathogenic
	c.302-1G>A	6	C	G	rs80358116	Pathogenic
BRCA2	c.475+1G>T	5	G	A	rs81002797	Pathogenic
	c.476-2A>G	6	A	G	rs81002853	Pathogenic
	c.4987-1G>C	17	C	T	rs730881495	Pathogenic/likely pathogenic
	c.682-2A>G	9	A	G	rs878853287	Pathogenic
	c.7008-2A>T	14	A	T	rs81002823	Pathogenic
	c.7617+1G>T	15	G	A	rs397507922	Pathogenic/likely pathogenic
	c.8487+1G>A	19	G	A	rs81002798	Pathogenic
	c.8953+1G>A	22	G	T	rs81002882	Pathogenic/likely pathogenic
	c.7618-1G>A	16	G	A	rs397507389	Pathogenic

## Discussion

This study sought to identify BRCA1 and BRCA 2 mutations among triple-negative breast cancer female patients in one of the major teaching and referral hospitals in Kenya, to inform an ongoing larger study on genetic mutation testing. To the best of our knowledge, diagnosis of breast cancer at the age of <15 years is very low, and our study could as well be among the first study to report genetic assessment based on BRCA1 and BRCA2 at this young age. From the study, pathogenic, likely pathogenic, VUS, and those with conflicting pathogenicity were identified. Such variations have been reported in other studies, further indicating the prospect of such findings [13]. Notably, most of the mutations identified (68.8%) in our study were of the pathogenic type. This indicates an increased risk of cancer and a likely predisposing factor for family members of the index patient. We detected one variant (c.5352delG: p. Leu1785Terfs) on exon 21 of BRCA1, that was not matching to any variant in the BRCA1 and BRCA2 Exchange and ClinVar databases. This variant was reported in a Sri Lankan population cohort in an international conference proceeding in 2019 [14]. This finding highlights the importance of performing genetic sequencing to detect emergence of new variants and their implication in patient management. It is estimated that 40%-80% of carriers of BRCA1 and BRCA2 mutations have a probability of developing breast cancer and increased risk is also associated with family history [15-17]. It is not known in our population, the extent of such mutations since this screening was carried out on a few individuals. No surveillance study has been conducted in Kenya toward this objective. The annual diagnosis of new breast cancer cases stands at 6000 cases in Kenya, which could hypothetically imply that the extent of BRCA1 and BRCA2 mutation is higher in our population and goes undetected due to lack of screening. Availability and accessibility of screening for BRCA1 and 2 among the most at risk would help timely detection and thereby early intervention in the population. Triple-negative breast cancers grow and spread faster, have fewer treatment options, and tend to have a worse prognosis. The extent of such patients in our population is likely to complicate management, considering our resource-limited settings. Since such tumors are unresponsive to endocrine therapy or human epidermal growth factor receptor 2 (HER2) -targeted treatment [18] they make survival time shorter and mortality rate higher [19]. The need for screening service is therefore a priority. It is estimated that TNBCs occur mostly among women aged below 40 years [20], and those with BRCA1 mutations. In the current study, three of the six women were below 40 years and the mutations detected were higher in BRCA1 than in BRCA2. Our findings are comparable to the findings of Lovejoy *et al*. 2020 [21]. In this study, all the patients had invasive ductal carcinomas as the histological BC subtype in both BRCA 1 and BRCA 2 mutation carriers. These findings are similar to those of Rajagopal *et al*. 2022 [22]. In addition, their report indicated that most tumors were grade II or III, findings that were similar to those of our study. Furthermore, other studies observed the highest prevalence of BRCA1 mutant tumors in triple-negative breast cancer patients [22,23] similar to our findings. Age at diagnosis among the participants in this study was another important factor to note. For all except one, the first diagnosis was within a year or two after noticing a breast lump, a factor which is good for the cancer program since it provides for early interventions and a possible increased survival rate. The exception here was one participant aged < 20 years, whose age at diagnosis was <15 years. This case was significant since younger women do not consider themselves at risk for breast cancer. Since their breast tissue is generally denser than the breast tissue in older women, this could mask early detection and screening programs are not often recommended at this early age. However, as indicated in our study, it may be important to focus on early age, especially where family history and genetic screening are indicative. Breast cancer accounts for more than 40% of all cancers in women below 40 years [24]. It is estimated that 7% of women with breast cancer have a diagnosis before the age of 40 years. All our study participants were within that age bracket at the time of diagnosis except two who were in the age bracket of 45-54 years.

**Limitations:** although this study had a small sample size, it is important to note that three participants had a family history of breast cancer, liver, pancreas, skin, and prostate cancers. This underlines the importance of genetic screening. Both family history and age are important risk factors for breast cancer [24,25]. Due to the high cost of genetic screening and resource constraints, only six TNBC patients were first tested for genetic mutations to inform the ongoing study. Among the mutations identified in the study one variant (c.5352delG: p. Leu1785Terfs) was not characterized since it did not match any in the BRCA Exchange and ClinVar databases, such may warrant further investigation on its association with breast cancer. While the mutations identified were common in at least five patients, further investigations including a large sample size should be considered to validate these findings.

**Funding:** this research was funded by National Research Fund, Kenya (Postgraduate grant 2016).

**Unanswered questions and future research:** most of the mutations identified were of the pathogenic type and were common among 5 participants. It is not known why the participants had common mutations, yet they were from different ethnic groups. Variants of undetermined significance were also identified. In addition, one variant could not be identified in the BRCA Exchange and ClinVar databases. Further research may be required to explore these variations.

## Conclusion

Findings from this study revealed the existence of BRCA1 and BRCA2 genetic mutations that could be common among our population. This data forms a basis for our ongoing prospective study on the extent of genetic mutation and patients’ characteristics among breast cancers in Kenya. This study also highlights a spectrum of pathogenic variants of BRCA gene mutations in our population. However, a study involving a larger cohort of breast cancer patients with or without a family history of cancer should be explored to validate these findings. A study on the variants of undetermined significance could also establish their clinical significance in breast cancer among our population.

### What is known about this topic

- *Mutations in BRCA1 and BRCA2 genes confer an increased lifetime risk for breast and ovarian cancer (71.4-87% for the BRCA1 mutation and 77-88% for the BRCA2 mutation) at the ages of 70-80 years;*
- *Patients can benefit from personalized therapy if risk identification is made earlier;*
- *Early identification enables women to proactively participate in high-risk screening programs, chemoprevention, and risk-reducing surgery*.

### What this study adds

- *The study has shown that we have common types of mutations in our setup that are highly likely to be associated with triple-negative breast cancer;*
- *The clinical relevance of the mutations identified has been demonstrated;*
- *A report on genetic assessment based on BRCA1 and BRCA2 among a minor is not common indicating the need for targeting such a young age group*.
